# The demethylase NMAD-1 regulates DNA replication and repair in the *Caenorhabditis elegans* germline

**DOI:** 10.1371/journal.pgen.1008252

**Published:** 2019-07-08

**Authors:** Simon Yuan Wang, Hui Mao, Hiroki Shibuya, Satoru Uzawa, Zach Klapholz O’Brown, Sage Wesenberg, Nara Shin, Takamune T. Saito, Jinmin Gao, Barbara J. Meyer, Monica P. Colaiácovo, Eric Lieberman Greer

**Affiliations:** 1 Division of Newborn Medicine, Children’s Hospital Boston, Boston, Massachusetts, United States of America; 2 Department of Pediatrics, Harvard Medical School, Boston Massachusetts, United States of America; 3 Howard Hughes Medical Institute and Department of Molecular and Cell Biology, University of California, Berkeley, Berkeley, California, United States of America; 4 Department of Genetics, Harvard Medical School, Boston, Massachusetts, United States of America; The University of Hong Kong, HONG KONG

## Abstract

The biological roles of nucleic acid methylation, other than at the C5-position of cytosines in CpG dinucleotides, are still not well understood. Here, we report genetic evidence for a critical role for the putative DNA demethylase NMAD-1 in regulating meiosis in *C*. *elegans*. *nmad-1* mutants have reduced fertility. They show defects in prophase I of meiosis, which leads to reduced embryo production and an increased incidence of males due to defective chromosomal segregation. In *nmad-1* mutant worms, nuclear staging beginning at the leptotene and zygotene stages is disorganized, the cohesin complex is mislocalized at the diplotene and diakinesis stages, and chromosomes are improperly condensed, fused, or lost by the end of diakinesis. RNA sequencing of the *nmad-1* germline revealed reduced induction of DNA replication and DNA damage response genes during meiosis, which was coupled with delayed DNA replication, impaired DNA repair and increased apoptosis of maturing oocytes. To begin to understand how NMAD-1 regulates DNA replication and repair, we used immunoprecipitation and mass spectrometry to identify NMAD-1 binding proteins. NMAD-1 binds to multiple proteins that regulate DNA repair and replication, including topoisomerase TOP-2 and co-localizes with TOP-2 on chromatin. Moreover, the majority of TOP-2 binding to chromatin depends on NMAD-1. These results suggest that NMAD-1 functions at DNA replication sites to regulate DNA replication and repair during meiosis.

## Author summary

Errors in meiosis are the leading cause of miscarriages, as well as developmental and intellectual disabilities. We have identified that NMAD-1, an enzyme which removes methyl moieties from nucleic acids, is essential for appropriate DNA damage response, DNA replication and meiosis in the nematode *C*. *elegans*. We have cytologically and genetically characterized the defects which occur due to deletion of NMAD-1 in the *C*. *elegans* germline. Additionally, we have begun to determine molecularly how NMAD-1 can regulate DNA replication, by demonstrating that NMAD-1 binds to components of the DNA replication machinery and is required for their appropriate localization to DNA. Characterizing how epigenetic modifications and the corresponding enzymes that add or remove epigenetic modifications can control the fundamental process of meiosis will have broad implications for understanding and eventually correcting errors in meiosis that disrupt normal development.

## Introduction

Meiosis is the process by which a diploid cell divides to produce haploid gametes. Errors at any stage during the carefully orchestrated events of meiosis can result in improper chromosome segregation and subsequent aneuploidy (cells with an abnormal number of chromosomes). Aneuploidy is detected in at least 20% of human pregnancies and is the most frequent chromosomal abnormality [1]. Although most errors in meiosis and aneuploidy lead to miscarriage, aneuploidy in fetuses that survive is the most common cause of both developmental and intellectual disabilities [2].

Methylation of nucleic acid bases increases the repertoire of information carried in a DNA or RNA sequence. The extent and diversity of recognized DNA and RNA methylation changes has expanded in recent years with the development of new and more sensitive detection technologies [3–6]. DNA methylation has been proposed to regulate self-versus-nonself recognition, distinguishing DNA damaged strands in need of repair, genomic imprinting, X-chromosome inactivation, transposon suppression, and transcription [4, 7, 8]. Meanwhile RNA methylation has been implicated in regulating translation initiation, RNA stability, RNA localization, and function [3, 5, 6, 9]. Understanding the biological importance of these modifications has been facilitated by identifying the methylases and demethylases that modify nucleic acids.

We and others previously identified methylation on the N6 position of adenines (6mA) as a novel DNA modification in metazoans [10, 11]. The AlkB family of dealkylating enzymes demethylates a diverse repertoire of methylated DNA and RNA in a variety of species [12–14]. There are nine AlkB family members in humans (Alkbh1-8 and FTO) and five AlkB family members in *C*. *elegans*. We identified the AlkB family member NMAD-1 (F09F7.7) as a dealkylating enzyme in *C*. *elegans* which demethylates 6mA and the DNA damage modification 3mC on DNA [10]. Moreover, 6mA was increased in *nmad-1* mutant worms as assessed by ultra-high performance liquid chromatography coupled with mass spectrometry (UHPLC-ms/ms). These results suggested that NMAD-1 is a 6mA DNA demethylase *in vivo*. Since our original publication multiple studies have identified 6mA DNA modifications in numerous eukaryotic species [11, 15–26] and that ALKBH4, the NMAD-1 homolog, demethylates 6mA *in vitro* [27]. In *C*. *elegans*, the level of 6mA on DNA has been shown to increase globally in response to mitochondrial stress and to mark genes important for stress response [26]. However, our recent work suggests that 6mA in DNA is difficult to measure and is less abundant than previously thought because of contamination with bacterial DNA, where 6mA is frequent, and because of the inability of current sequencing methods to accurately identify 6mA [28]. Because these DNA methylation events in eukaryotes are rare, it is still uncertain whether NMAD-1 functions *in vivo* as a 6mA and 3mC DNA demethylase. At this time, we cannot accurately map 6mA modifications in eukaryotes. In the absence of such a technique, we nonetheless wanted to investigate the role of NMAD-1 by characterizing *nmad-1* mutant worms. We previously found that mutation of NMAD-1 reduced fertility in *C*. *elegans* by an unknown mechanism [10]. Our goal here was to examine how *nmad-1* deficiency leads to infertility.

## Results

**Fig 1:**
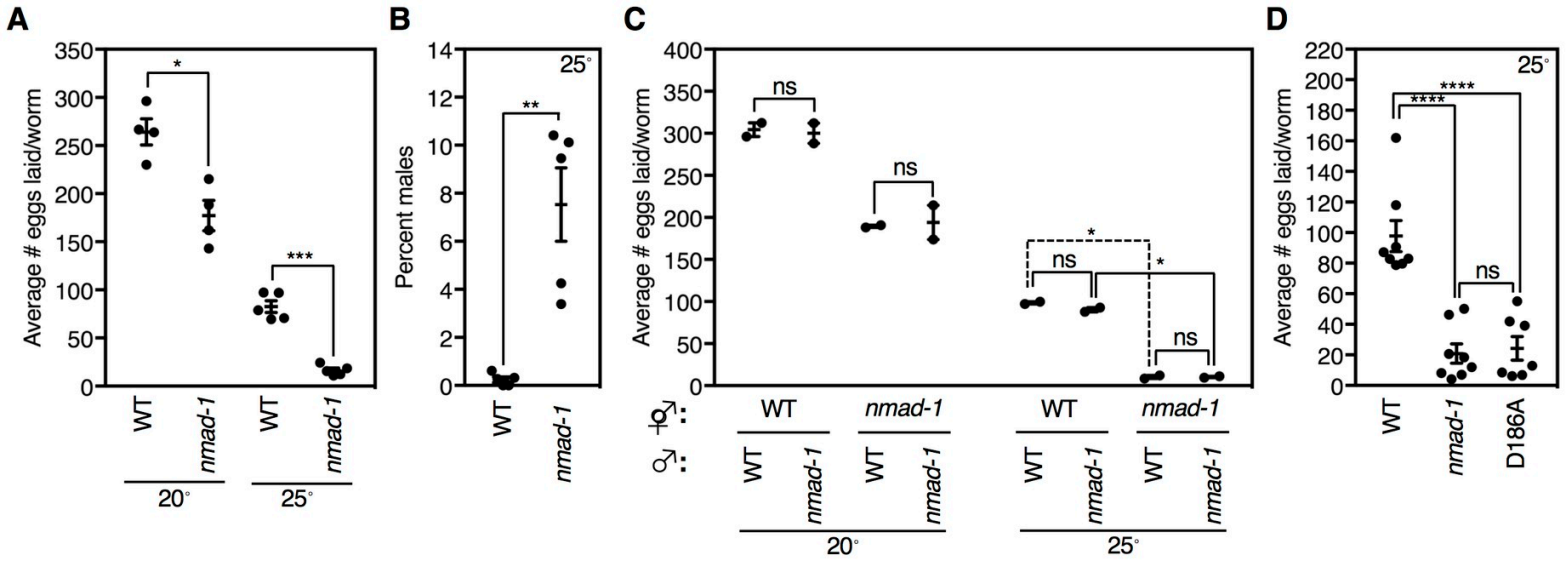
*nmad-1* mutants display sterility and evidence of X chromosome nondisjunction. A) *nmad-1* mutants lay fewer eggs than WT worms at 20° and 25°. This graph displays the mean ± SEM of four independent experiments: each experiment consists of average eggs laid for ten worms of each genotype performed in triplicate. B) *nmad-1* mutant worms have an increased incidence of males. This graph displays the mean ± SEM of four independent experiments: each experiment consists of average eggs laid for ten worms of each genotype performed in quadruplicate at 25°. Paired t-test were performed to compare WT and *nmad-1* male percentages. C) Fertility defects are maternally driven. WT or *nmad-1* mutant hermaphrodites were crossed with WT or *nmad-1* males. This graph displays the mean ± SEM of two independent experiments: each experiment consists of average eggs laid for ten worms of each genotype performed in triplicate or quintuplicate. Comparative statistics represent paired t tests. D) A mutation of the catalytic domain of NMAD-1, converting aspartic acid 186 to alanine (D186A), is sufficient to cause reduced fertility. This graph displays the mean ± SEM of seven independent experiments: each experiment consists of average eggs laid for ten worms of each genotype performed in triplicate at 25°. Paired t-test were performed to compare brood sizes. ns: not significant, * p<0.05, ** p<0.01.

In *C*. *elegans*, the rate of reproduction is controlled by a variety of factors, including food availability, developmental defects including vulva maturation, and meiotic errors [29–31]. To examine NMAD-1’s role in reproduction, we performed a detailed phenotypic and cytological analysis of an *nmad-1* deletion strain which removes greater than 95% of the AlkB domain. We first confirmed that *nmad-1* worms laid fewer eggs than wildtype worms at 20°C and found that the egg laying defect was exacerbated at 25°C (Fig 1A). *C*. *elegans* are typically hermaphrodites (XX) with a small number of males (XO) arising periodically (~ 0.01–0.3%) [32]. *nmad-1* mutant worms also showed a ~30-fold increased incidence of males at 25°C (Fig 1B). *C*. *elegans* have 5 autosomes and 1 sex chromosome. Males (XO) arise from a meiotic non-disjunction event during hermaphrodite (XX) meiosis that causes loss of one X chromosome [32]. To determine whether infertility was due to errors in the maternal or paternal lineage, we crossed wild type (WT) males with *nmad-1* hermaphrodites and *nmad-1* males with WT hermaphrodites. The paternal genotype had no effect on fertility (Fig 1C), suggesting that this is a maternally driven phenotype. To determine whether reduced fertility was due to NMAD-1’s demethylase activity, we examined the effects on fertility of mutation of aspartic acid 186 in the catalytic domain of NMAD-1, a residue homologous to aspartic acid 135 in bacterial AlkB, which is responsible for selectively binding nucleotides in the catalytic site [33]. We previously demonstrated that this mutation eliminated NMAD-1 catalytic activity *in vitro* [10]. *nmad-1 (D186A)* mutant worms had reduced fertility and an increased incidence of males that was similar to *nmad-1* worms (Fig 1D and S1A Fig). Thus fertility and achieving accurate X chromosome segregation depend on maternal NMAD-1 dealkylating activity.

**Fig 2:**
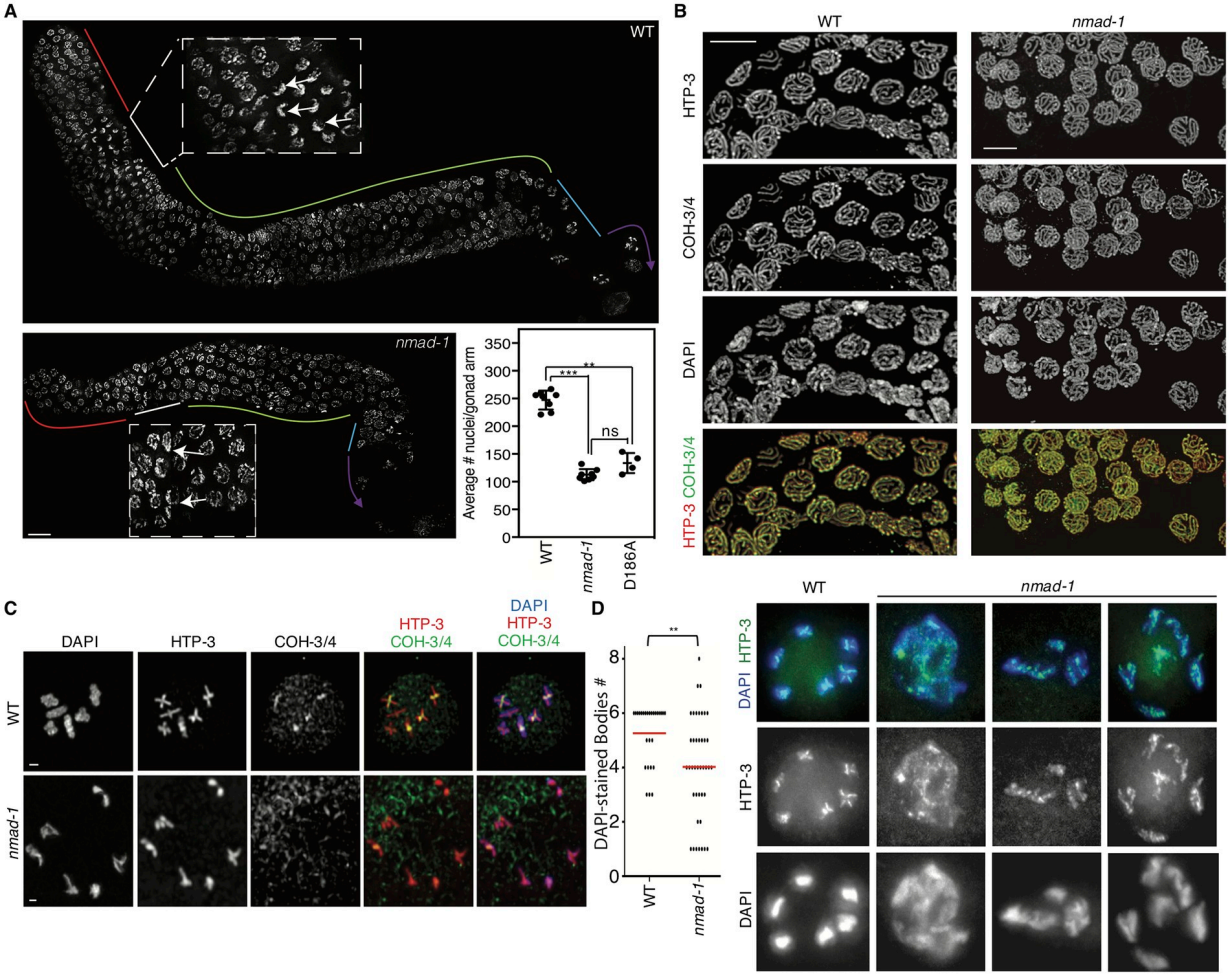
*nmad-1* mutant germlines are short, disorganized, and display chromosome loss or condensation defects by the end of prophase I. A) *nmad-1* germlines are compacted and have fewer nuclei relative to WT germlines and display intermixing of transition zone and pachytene stage nuclei as assessed by DAPI. Different zones are marked by color (pre-meiotic: red, transition zone: white, pachytene: green, diplotene: blue, diakinesis: purple). Scale bars are 10 microns. The inset displays average # of nuclei scored per halfway projection of each gonad arm. Mann-Whitney tests display significant differences between WT and *nmad-1* deletion or catalytic mutant strains. 4–8 germlines were quantified per genotype. B) Pachytene nuclei in *nmad-1* germlines are comparable to WT germlines as assessed by DAPI, HTP-3, and COH-3/4 staining. HTP-3 is shown in red, and COH-3/4 in green. Scale bars are 5 microns. C) By the diakineses stage COH-3/4 sometimes marks the short arm of cruciform structures in *nmad-1* mutants as in WT worms but sometimes fails to mark these regions. Displayed are representative images with DAPI shown in blue, HTP-3 in red, and COH-3/4 in green. Scale bars are 1 micron. D) *nmad-1* mutant worms have abnormal chromosome number and compaction at the diakinesis stage as visualized by DAPI for DNA and HTP-3 a protein localized to the axes of meiotic chromosomes. Representative images are shown on the right panel and a compilation of 27–41 nuclei are quantified in the left panel. ns: not significant, * p<0.05, ** p<0.01, *** p<0.0005.

As the oocyte matures, it progresses from the distal end of the gonad, termed the premeiotic tip, towards the vulva. Each stage of meiosis is partitioned spatially along the gonad, and different stages of meiosis can be identified by DAPI staining of nuclei which mark the dynamic changes in chromosome structure and organization during meiosis [34]. The *C*. *elegans* germline is a syncytium consisting of developing germ cell nuclei sharing a common cytoplasm. The distal end of the gonad, termed the premeiotic tip, contains germ cells which undergo mitosis. Following DNA replication at the premeiotic tip, nuclei then proceed through meiosis, first entering prophase I, which is subdivided in a spatiotemporal manner along the gonad arm into leptotene, zygotene, pachytene, diplotene, and diakinesis stages. The leptotene and zygotene stages occur in a region termed the transition zone where chromatin is positioned in the periphery of the nucleus acquiring a crescent shape appearance. This is also the stage at which programmed meiotic DNA double strand breaks (DSBs) start to form, homologous chromosomes pair, and synapsis is initiated between homologs. After synapsis has been completed, the chromosomes redistribute to the nuclear periphery in the pachytene stage. During the pachytene stage, crossover recombination is completed and by late pachytene the synaptonemal complex begins to disassemble. As homologous chromosomes progress through diplotene into diakinesis, they begin to condense, and at diakinesis homologs are held together through chiasmata as a result of the single crossover event that takes place earlier in prophase between each pair of homologs, underpinned by flanking sister chromatid cohesion. Crossovers result in the reciprocal exchange of genetic material between homologs to promote genetic diversity and the formation of physical links between homologs (chiasmata) required for proper chromosome alignment at the metaphase plate and subsequent accurate segregation to opposite ends of the spindle at meiosis I. We first examined the gonad arms of *nmad-1* mutant worms, raised from the L4 larval stage at 25°C, where infertility is greater. Although the number and morphology of premeiotic nuclei were similar between WT and *nmad-1* mutant worms, the gonad arms of *nmad-1* mutant worms were significantly smaller and had fewer nuclei (Fig 2A, p<0.005). In the *nmad-1* mutant there was an intermixing of transition zone nuclei and pachytene stage nuclei as defined by their peripheral chromatin morphology. The pachytene zone was also significantly compacted compared to WT germlines (Fig 2A and 2B). To determine whether chromosome synapsis occurred properly and axis morphogenesis is normal at the pachytene stage, we stained for HTP-3, an axial component of the synaptonemal complex that forms between paired homologous chromosomes [35, 36]. As in WT, HTP-3 formed typical tracks along the lengths of the chromosomes at the pachytene stage in *nmad-1* mutant germlines (Fig 2B). Sister chromatids are held together by the cohesin complex beginning during DNA replication, which we visualized by staining for COH-3/4. Although COH-3/4 staining in *nmad-1* oocytes was similar to WT oocytes at the pachytene stage (Fig 2B), COH-3/4 did not properly localize to the short arms of bivalents during diakinesis as it does in wildtype worms (Fig 2C). To determine whether crossover designation was defective in *nmad-1* mutant worms, we examined COSA-1, a cyclin-like protein involved in designating which DSBs will be converted into a single crossover along each homolog pair [37]. COSA-1 is detected as six distinct foci per nucleus in both WT and *nmad-1* mutant worms from late pachytene to diplotene (S1B Fig) and diakinesis (S1C Fig) stages, suggesting that crossover designation was intact in *nmad-1* mutant worms. However, *nmad-1* mutant worm oocytes showed striking chromatin disorganization at diakinesis, which was exacerbated if the parent was cultured at 25°C from the egg stage, which caused complete sterility. While 6 bivalents were detected in WT oocytes at diakinesis, fewer bivalents and less condensed chromatin were apparent in oocytes in *nmad-1* mutant germlines (Fig 2D). In WT oocytes, the chromatin of each bivalent was clearly separated from the others, while in *nmad-1* nuclei at diakinesis some of the chromosomes appeared fused, some were improperly condensed, and sometimes fewer than 6 DAPI-stained bodies were observed. These data suggest that *nmad-1* is essential for appropriate meiosis, and its deficiency leads to major defects in chromosomal condensation and organization evident at diakinesis.

**Fig 3:**
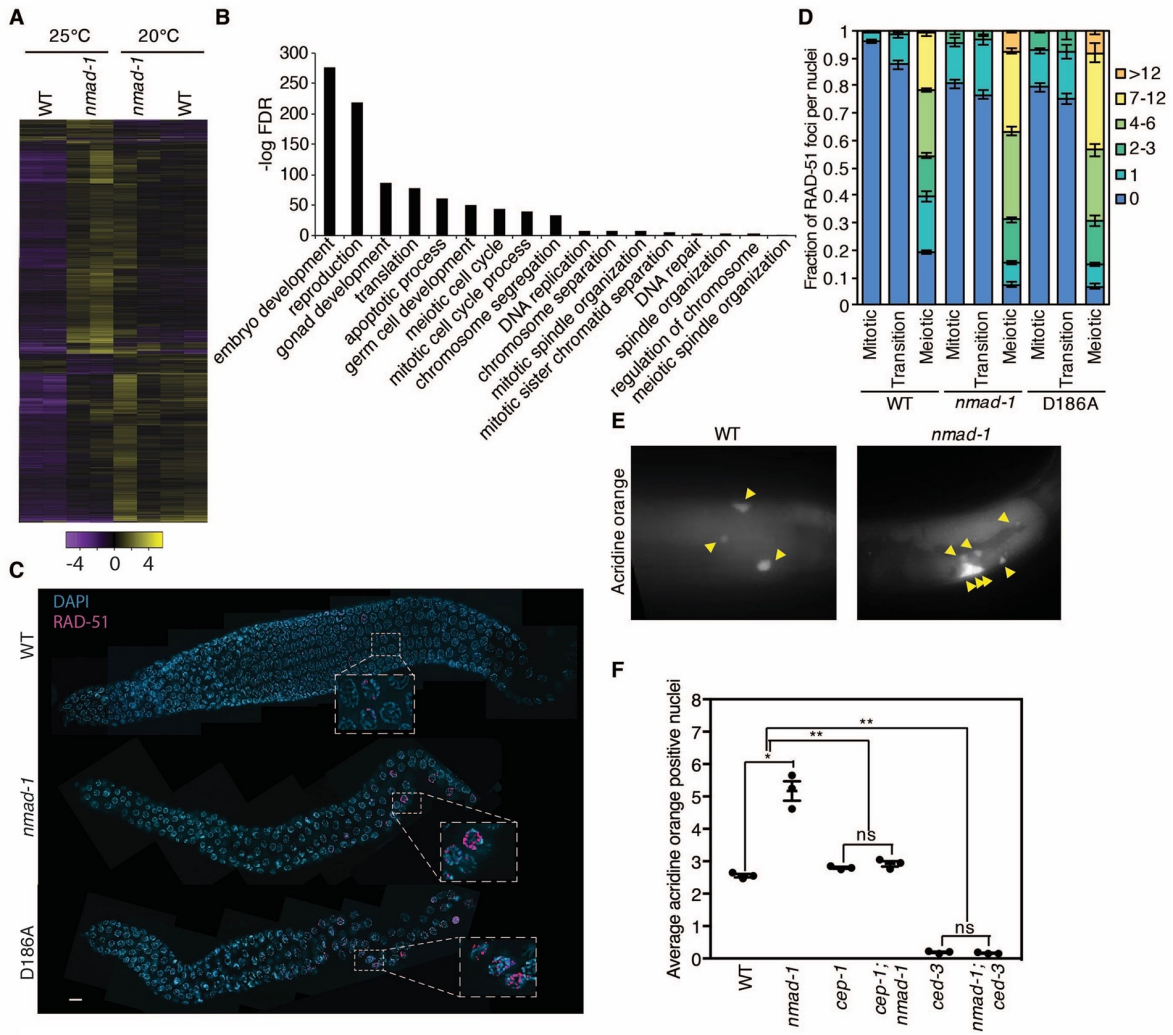
*nmad-1* mutant germlines have gene expression defects in DNA damage and replication pathways and display elevated DNA damage. A) Hierarchical clustering of RNAseq data from extracted germlines of WT and *nmad-1* mutant worms grown at 20° and 25° reveal gene expression differences that are most apparent at 25°. B) The top gene ontology (GO) categories of down-regulated genes in *nmad-1* mutant worms at 25° are enriched for genes regulating reproduction, gonad development, apoptosis, DNA replication, and DNA repair. To determine whether *nmad-1* mutants display defects in these processes, we first examined *nmad-1* developmental progression. *nmad-1* mutant worms display a slight delay in development at 20° which is more apparent at 25° (S3 Fig). C) RAD-51 staining of dissected whole mounted worm germlines show elevated RAD-51 foci number and persistence in *nmad-1* and D186A transgenic mutant worms compared with WT worms. DAPI staining in blue and RAD-51 staining in red. Scale bars are 10 microns. D) Quantification of RAD-51 foci in 4–8 germlines from WT, *nmad-1*, and D186A mutant worms demonstrates increased RAD-51 foci arising earlier, being more plentiful, and persisting longer in *nmad-1* mutant worms. E) There is increased apoptosis in the *nmad-1* mutant germline as assessed by acridine orange. Yellow arrows point to apoptotic nuclei. Images were taken of the gonadal loop region as developing oocytes transition from the pachytene to the diplotene stage. F) Increased apoptosis in *nmad-1* mutant worms is dependent on the p53 homolog *cep-1* and the caspase 1 homolog *ced-3* as assessed by quantification of apoptosis occurrence in WT, *nmad-1*, *cep-1*, *cep-1*:*nmad-1*, *ced-3*, and *nmad-1;ced-3* mutant worms by acridine orange. This graph represents the mean ± SEM of three independent experiments: each experiment consists of apoptosis measurements of 15–20 worm germlines per genotype examined at 25°. Individual genotypes were compared by paired t tests while the interaction of genotypes was analyzed by two-way ANOVA. ns: not significant, * p<0.05, ** p<0.01).

To begin to understand why *nmad-1* deletion causes defects in chromosome morphogenesis, we set out to identify genes that might be misregulated in the germline of *nmad-1* mutant worms. We performed RNA-seq on germlines extracted from WT and *nmad-1* mutant worms at 20°C and 25°C (Fig 3A and S1 Table). While germline gene expression in *nmad-1* and WT worms was significantly different at 20°C, this difference was further exacerbated at 25°C (Fig 3A, S2A and S2B Fig). A gene ontology (GO) analysis of the differentially regulated genes at 25°C in the *nmad-1* mutant worms revealed enrichment of genes involved in reproduction, gonad development, apoptosis, meiosis, DNA replication and DNA repair (Fig 3B, S2C and S2D Fig, and S1 Table). The majority of these genes were downregulated, or failed to be upregulated, in the *nmad-1* mutant germline (S2C and S2D Fig). A comparison of the differentially regulated genes at 25°C in the germlines of *nmad-1* mutant worms compared with germline enriched genes in WT [38] similarly revealed an up regulation of some genes involved in reproduction and a down regulation of other genes involved in reproduction, apoptosis, and germ cell development (S2E and S2F Fig), suggesting that these differentially regulated gene categories were not simply a consequence of comparing germline enriched genes to all genes.

Because *nmad-1* mutant worms induce DNA damage repair genes to a lesser extent than WT worms, we next examined whether *nmad-1* mutant worms were defective in DSB repair progression. The nuclease SPO-11 generates programmed meiotic DSBs and DSB repair progression, as visualized by RAD-51 immunostaining. RAD-51 foci are first detected in nuclei at the transition zone [39], where we identified an intermingling of transition zone and pachytene nuclei. During DSB repair, replication protein A (RPA) is recruited initially to DSBs to protect resected DNA ends and is then replaced by RAD-51, which facilitates strand invasion during homologous recombination [40]. Both *nmad-1* deletion and *nmad-1(D186A)* mutants displayed spatially premature and persistent RAD-51 foci, occurring as early as the mitotic zone, that accumulated by the late pachytene stage of meiosis when RAD-51 foci are mostly absent in WT worms (Fig 3C and 3D). Normally at the mid to late diplotene stage DSB repair switches from homologous recombination to non-homologous end joining (NHEJ) repair and is therefore no longer dependent on RAD-51 [34]. The elevation in RAD-51 foci found in *nmad-1* mutants did not persist past pachytene. RAD-51 foci were absent from oocytes at diakinesis of both wild-type and *nmad-1* animals, (S4A Fig), suggesting that the transition to NHEJ repair occurs normally in *nmad-1* mutants. The elevated levels of RAD-51 foci in *nmad-1* mutant worms disappeared in *spo-11;nmad-1* double mutant worms, which did not have any RAD-51 foci, suggesting that the formation of RAD-51 foci depended on DSBs formed by SPO-11 (S4B Fig). Since SPO-11 is a meiosis-specific nuclease, this result suggests that the DSBs and the defect in their repair observed in *nmad-1* mutant worms arise during meiosis rather than mitosis. However, we also detected SPO-11-dependent RAD-51 foci in the mitotic region of *nmad-1* mutant worms (S4B Fig), which suggests that the switch between mitotic and meiotic programs could be dysregulated or that the normal spatial separation between these stages could be disrupted, leading to intermixing of cells at different stages. When *spo-11* was mutated in the *nmad-1* mutant worms, ~12 univalents were observed in the majority of diakinesis nuclei at both 20°C and 25°C, indicating that the disorganization in the *nmad-1* mutant worms is dependent on *spo-11* suggesting that it is a consequence of aberrant meiotic recombination (S4C Fig). Together, these results suggest that DSBs are properly induced during meiosis, but fail to be repaired efficiently in *nmad-1* mutant worms and that NMAD-1 regulates normal DNA damage repair during meiosis.

Several observations suggested that apoptosis might be increased in the germlines of *nmad-1* mutants: (1) expression of genes involved in apoptosis were misregulated (Fig 3B and S2D–S2H Fig), (2) levels of RAD-51 foci were increased in the germline, a sign of increased unrepaired DNA damage (Fig 3C and 3D), and (3) few eggs were laid by *nmad-1* mutant worms (Fig 1). To assess cell death, WT and *nmad-1* mutant worms were stained either with acridine orange, which labels apoptotic nuclei [41], or crossed with worms expressing a GFP::CED-1 transgene that identifies apoptotic cells for phagocytosis [42]. *nmad-1* mutant worms had increased germ cell apoptosis compared with WT worms at 25°C as assessed by both counting acridine orange positive nuclei (Fig 3E) and CED-1::GFP fluorescent apoptotic germ cells (S4D Fig). Increased cell death was dependent on the caspase *ced-3* [43], suggesting that cell death was mediated by classical apoptotic signaling (Fig 3F and S4E Fig). Increased apoptosis was also dependent on the p53 homolog *cep-1*, suggesting that apoptosis was triggered by unrepaired DNA damage [44] (Fig 3F and S4E Fig). Together these results suggest that developing oocytes in *nmad-1* mutant worms undergo apoptosis because of defective DNA damage repair in the germline.

**Fig 4:**
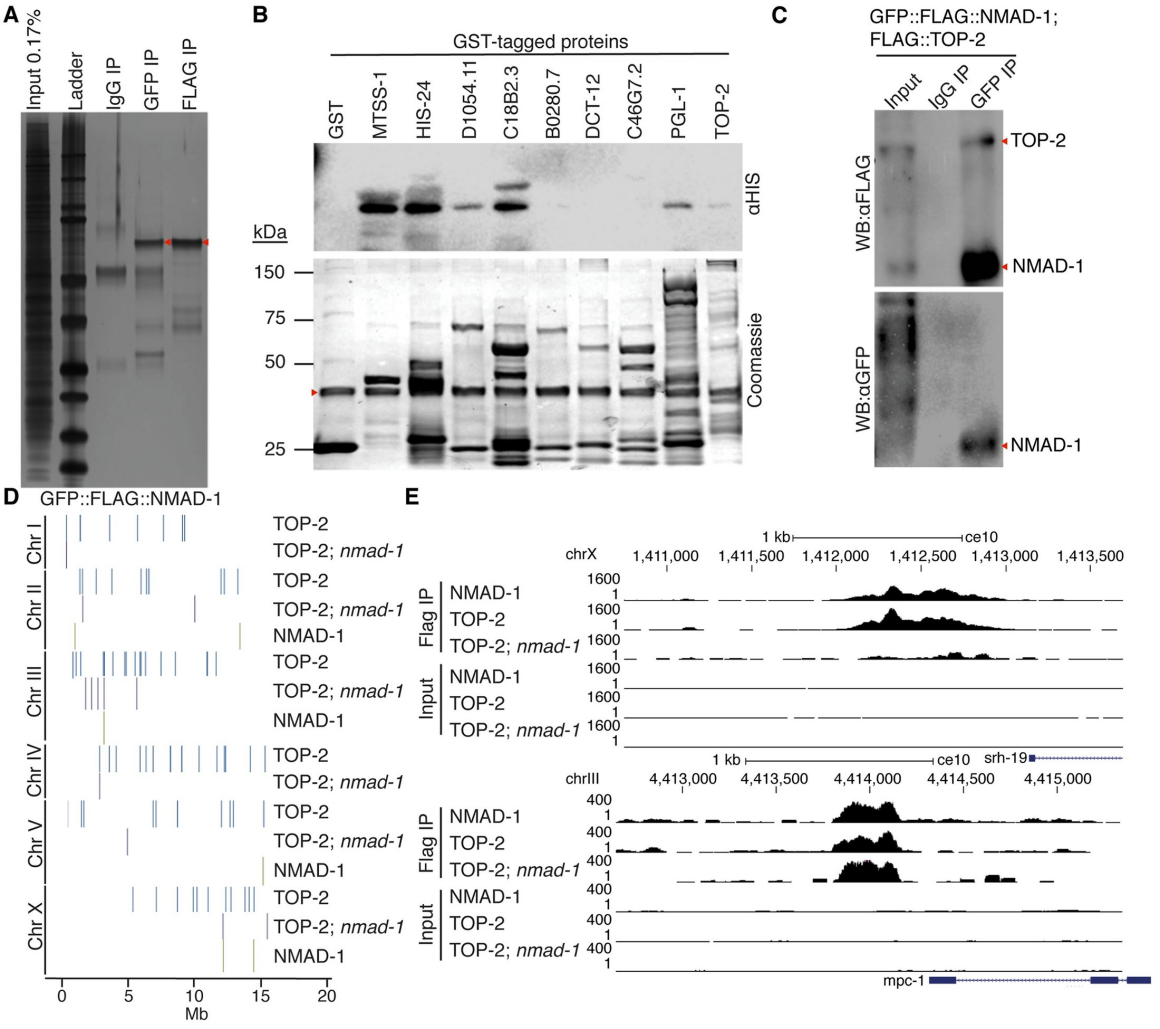
NMAD-1 interacts with DNA replication and repair machinery. A) Silver stained SDS-page gel of GFP:FLAG:*nmad-1* transgenic strain reveals unique NMAD-1 binding partners. Control IPs were performed with IgG. Each lane was submitted to mass spectrometry and this gel is representative of three independent experiments. Red arrow indicates GFP:FLAG:NMAD-1. B) *In vitro* binding assays confirm interactions of several NMAD-1 interacting proteins including components of the replication machinery identified by mass spectrometry. HIS:NMAD-1 was incubated with GST tagged interacting proteins. Upper blot is probed with HIS antibody and lower blot is coomassie stained control blot. Red arrow indicates HIS:NMAD-1. C) IP of GFP confirms NMAD-1 *in vivo* binding to TOP-2 in double transgenic strain GFP::FLAG::*nmad-1; top-2*::*FLAG*. IgG or GFP antibodies were used to IP from double transgenic strain and western blots were probed with GFP (bottom blot) or Flag (top blot) antibodies. 1% of the input is loaded as a control. D) ChIPseq of NMAD-1 and TOP-2 in GFP::Flag::*nmad-1* and *top-2*::*Flag* transgenic strains with or without *nmad-1* reveals that TOP-2 and NMAD-1 bind to the same locations in the genome and greater than half of TOP-2 binding is dependent on *nmad-1*. E) Representative ChIPseq profiles of TOP-2 bound genes that are *nmad-1* dependent (top panel) or independent (lower panel).

To begin to understand how NMAD-1 deficiency leads to this complex germline phenotype, we generated a transgenic strain that expressed a Flag-tagged GFP::NMAD-1 fusion protein and isolated and identified candidate NMAD-1 interacting proteins by GFP and Flag immunoprecipitation (IP), compared to control IgG antibody IP, followed by mass spectrometry (Fig 4A and S2 Table). NMAD-1 interacting peptides corresponding to 136 unique proteins were retrieved in the GFP IP, which were not in the control IP. 69 proteins were retrieved in the FLAG IP, which were not in the control IP. 43 of these proteins were identified in both pull downs. 12 putative binding proteins were prioritized for experimental testing based on their presence in replicate IP experiments and whether they might be involved in DNA repair (MTSS-1 and TOP-2), DNA replication (MTSS-1, MCM-4, and TOP-2) or apoptotic phenotypes that we identified in meiotic oocytes of *nmad-1* mutant worms. To test whether these candidate NMAD-1 binding proteins bound directly to NMAD-1, we performed *in vitro* binding assays using recombinant His-tagged NMAD-1 and GST-tagged or untagged candidate binders (Fig 4B and S5 Fig). NMAD-1 directly bound to MTSS-1, TOP-2, and MCM-4, components of the DNA replication machinery [45–48]. MTSS-1 is a single strand DNA binding protein which is critical for DNA replication, recombination and repair. MCM-4 is a component of the minichromosome maintenance complex which is responsible for licensing origins for DNA replication and is the DNA helicase complex responsible for unwinding the DNA at the origins of replication [46, 49]. TOP-2 is a topoisomerase involved in transient dsDNA breaks to facilitate DNA unwinding in advance of DNA replication, chromosome segregation, and transcription [50]. To test whether NMAD-1 also bound to these DNA replication proteins *in vivo*, we crossed strains carrying tagged *mtss-1*, *top-2*, and *mcm-4* transgenes with our tagged NMAD-1 strain and performed IPs followed by immunoblot. Although we were unable to confirm NMAD-1’s interaction with MTSS-1 and MCM-4 *in vivo*, we confirmed that NMAD-1 immunoprecipitated specifically with Flag-tagged topoisomerase II (TOP-2) *in vivo* [45] (Fig 4C), suggesting that NMAD-1 binds to TOP-2 in cells. Interestingly, *top-2* mutant worms display defects in meiotic chromosome segregation [45].

If NMAD-1 binds to TOP-2, we reasoned that they might co-localize on chromatin. We therefore performed chromatin immunoprecipitation followed by sequencing (ChIPseq) with Flag antibody on *flag*::*top-2* and *flag*::*GFP*::*nmad-1* transgenic and wildtype control strains (S3 Table). Common peaks in biological replicate ChIPseq experiments were identified using Irreproducibility Discovery Rate IDR (2.0.2). After removal of blacklist regions from modENCODE [51] and regions that were enriched in our input flag control samples we identified 77 high confidence TOP-2 binding sites and 7 high-confidence NMAD-1 binding sites. 6 of the 7 NMAD-1 bound regions were co-bound by TOP-2 (Fig 4D, p = 0.0047 by Fisher’s exact test), suggesting that NMAD-1 and TOP-2 co-localize on DNA. To determine whether TOP-2 binding was dependent on NMAD-1, we crossed the *flag*::*top-2* strain with the *nmad-1* deletion strain and performed ChIPseq of TOP-2 with the Flag antibody. *nmad-1* deletion caused TOP-2 to be removed from 3 of the 6 NMAD-1 TOP-2 co-bound sites and 71 of the 77 high confidence TOP-2 sites as well as relocalized TOP-2 to another 6 new sites (Fig 4D and 4E). These results suggest that TOP-2 and NMAD-1 interact on chromatin and that NMAD-1 regulates TOP-2 localization to most sites.

**Fig 5:**
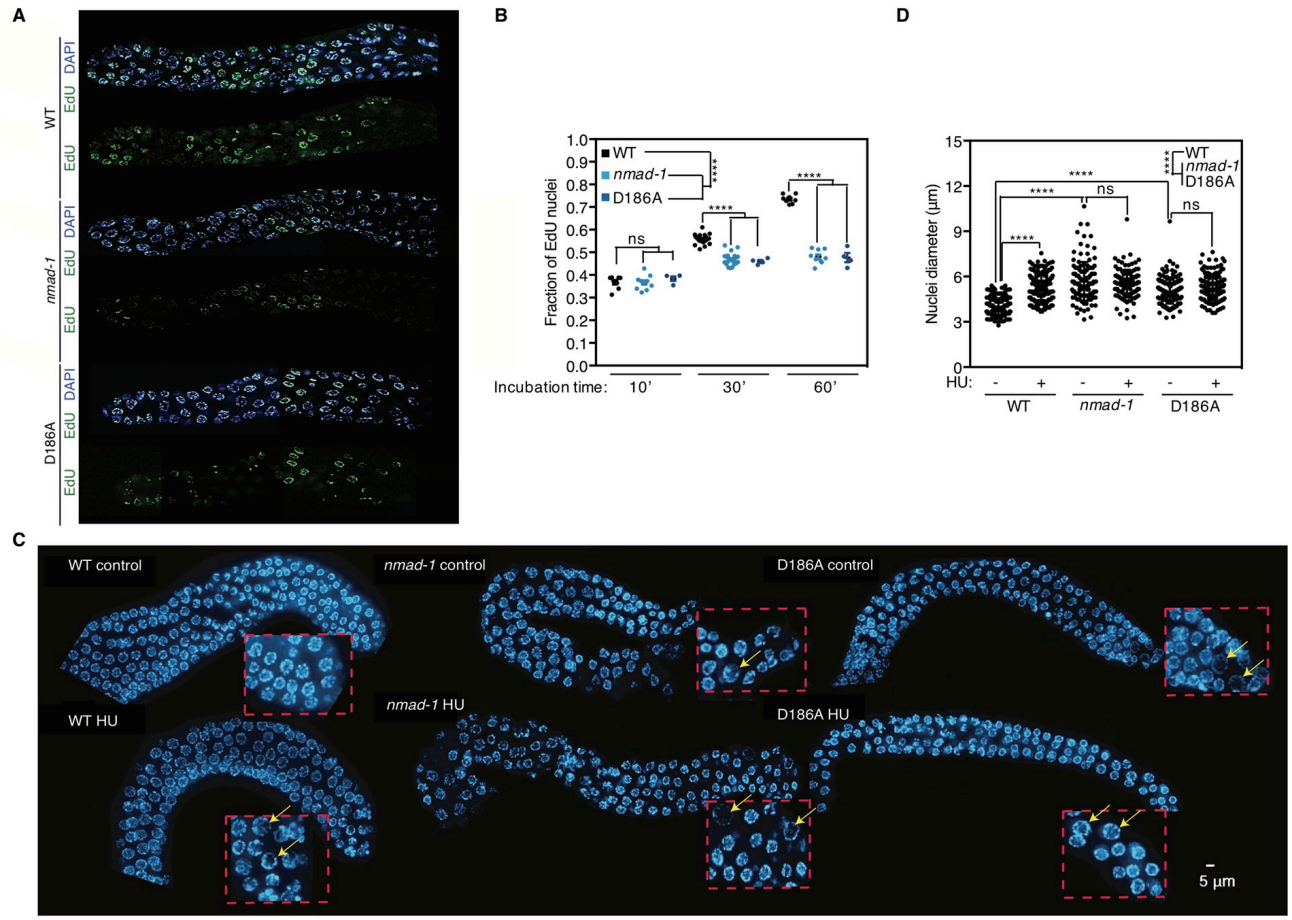
*nmad-1* mutant worms have delayed DNA replication rates. A) Replication rate is slowed in *nmad-1* and D186A mutant worms as assessed by EdU incorporation in the germline. Representative images are shown with DAPI stain in blue and EdU in green. B) A quantification of the fraction of EdU positive nuclei in 4–23 germline after EdU incubation for 10, 30, or 60 minutes. 2-way ANOVA reveals that *nmad-1* and D186A mutant worms have a significantly different rate of EdU incorporation compared with WT worms. Individual time comparisons were compared by Welch’s unpaired t test. C) *nmad-1* and D186A mutants showed enlarged nuclei comparable to wildtype worms and hydroxyurea treatment increases wildtype worm nuclei but has no effect on the already enlarged nuclei of *nmad-1* and D186A mutant worms. Yellow arrows highlight the swelling nuclei in zoomed-in squares at premeiotic tips. D) Quantification of the nuclei diameter in a compilation of 116 to 226 nuclei for each groups. Ns: not significant, *** p<0.001, **** p<0.0001. Scale bars are 5 microns.

Because of the reduced embryo production (Fig 1), disordered progression through meiosis, defective expression of DNA replication genes (Fig 3), and alteration in TOP-2 binding to chromatin in the germline in *nmad-1* mutants (Fig 4D), we predicted that *nmad-1* mutants would have delayed or defective DNA replication. We therefore used EdU incorporation to compare the DNA replication rate in the developing germline of WT, *nmad-1* deletion and *nmad-1(D186A)* catalytic mutant worms. Both the deletion and catalytic mutation significantly reduced EdU incorporation (Fig 5A and 5B). Reduced EdU labeling could be due to either delayed DNA replication or a reduced number of nuclei undergoing mitosis. Although *nmad-1* mutant worms have a reduced pachytene zone (Fig 2A), their premeiotic region had the same number of nuclei (p = 0.1475 by repeated measure one-way ANOVA with Geisser-Greenhouse correction). Moreover, reduced nuclei number is unlikely to be the cause of reduced EdU incorporation as EdU incorporation at the start of feeding was indistinguishable between the WT and mutant strains (Fig 5B), suggesting that the number of cells undergoing mitosis was similar. To measure mitotic cells directly, we examined levels of phosphorylated Histone H3 at Serine 10 (H3pS10), which is phosphorylated at the end of the G2 phase and removed by the telophase stage at the end of the mitotic phase [52, 53], and found similar levels of H3pS10 in WT, *nmad-1* deletion, and *nmad-1(D186A)* catalytic mutant worms (S6 Fig). Altogether, these data therefore suggest that *nmad-1* mutant worms have a defect in DNA replication. To independently examine DNA replication in *nmad-1* mutant worms, we treated WT, *nmad-1* deletion, and *nmad-1(D186A)* catalytic mutant worms with hydroxyurea (HU), an inhibitor of ribonucleotide reductase which causes replication fork stalling leading to enlarged nuclei in the premeiotic tip of *C*. *elegans* germlines due to continued cellular growth in the absence of cell division [54]. By performing this genetic experiment, we wanted to determine whether *nmad-1* inhibition further exacerbated the S-phase arrest. We found that HU treatment caused enlarged nuclear diameters in WT worms but did not further enlarge the already enlarged nuclear diameters of *nmad-1* deletion and *nmad-1(D186A)* catalytic mutant worm germline nuclei (Fig 5C and 5D). Together these data suggest that *nmad-1* mutant worms display defects in DNA replication.

## Discussion

Here we show that the putative demethylase NMAD-1 plays a critical role in DNA replication and DNA damage repair during meiosis in *C*. *elegans*. NMAD-1 binds to the same sites on chromatin in the germline as TOP-2 and physically interacts directly with TOP-2 both *in vitro* and *in vivo*, suggesting that NMAD-1 acts on chromatin to regulate these processes in meiosis. Both the *nmad-1* deletion and catalytic mutant strains have impaired fertility, an increased incidence of males, elevated RAD-51 foci, and reduced replication rates (Figs 1D, 3C and 5 and S1A Fig). These findings suggest that NMAD-1’s demethylation activity at DNA is central to its role in meiosis.

We previously identified NMAD-1 as a 6mA DNA demethylase [10] and it has been proposed as a conserved 6mA DNA demetylase in the silkworm *Bombyx mori* [55] as well as in mice [27]. However, because of the low levels of 6mA DNA methylation in *C*. *elegans* (which appear to be even lower than initially reported [28]), we have been unable to determine whether 6mA DNA is the NMAD-1 substrate in *C*. *elegans* responsible for its profound effect on meiosis. Additional accurate sequencing technologies to map and quantify 6mA at single-base resolution in gDNA will need to be developed to reveal whether modifications to DNA are present and biologically significant for the role of NMAD-1 in regulating meiosis. Future studies will need to determine the NMAD-1 substrate that regulates DNA repair and replication in meiosis, which could be 6mA or 3mC DNA, or an unidentified other nucleic acid or even protein substrate.

In prokaryotes, 6mA regulates DNA replication by marking the origin of replication and hemimethylation of the origin delays DNA replication [56, 57]. One possibility is that, in worms, 6mA marks origins of replication and needs to be removed by NMAD-1 for DNA replication to proceed. In any event, a defect in the NMAD-1 demethylase strongly disrupts replication, DSB repair, and chromosome segregation in meiosis, ultimately leading to embryo death and sterility. Deficiency of ALKBH4, the closest mammalian homolog to NMAD-1, has been reported to cause reproduction defects in mice and zebrafish [58, 59], suggesting that this dealkylating enzyme might play a conserved role in regulating DNA replication throughout eukaryotes. Together our findings reveal a new layer of regulation where this demethylating enzyme is required to physically interact with the DNA repair and DNA replication machinery on the chromatin to facilitate appropriate meiosis progression.

## Materials and methods

### Strains used

The N2 Bristol strain was used as the WT background. The following mutations were used in this study: LG1: *cep-1(lg12501)*, LGIII: *nmad-1(ok3133)*, *nmad-1(D186A)*, LGIV: *spo-11(ok79)*, *ced-3(n717)*. Transgenes: bcIs39[*P*_*lim-7*_:*ced-1*::*gfp*], *gfp*::*3xflag*::*nmad-1*, and *top-2*::*3xflag(av64)*. Some of these worm strains have been previously characterized [10, 39, 45]. Worms were grown on *dam*^-^*dcm*^-^ bacteria (NEB C2925) in all experiments.

### Antibodies

Antibodies were used at the following dilutions: rabbit α-RAD-51 (SDIX, 1:200), rabbit α- H4K20me1 (Ab9051, 1:200), rabbit α-GFP (A11122), α-mouse FLAG (F3165, 1:2000), α-mouse HIS (Millipore, 05–949, 1:2000), α-mouse GST (Millipore 05–311, 1:2000), rabbit α-Cy3 (Jackson ImmunoResearch Laboratories, 1:200), rabbit α-FITC (Jackson ImmunoResearch Laboratories, 1:200), mouse α-Cy3 (Jackson ImmunoResearch Laboratories, 1:200), guinea pig α-Cy5 (Jackson ImmunoResearch Laboratories, 1:200), α-H3S10p (Millipore 06–570, 1:200). DAPI (Sigma, 1 μg/ml) was used to counterstain DNA.

### Egg laying assay

Ten staged L4 worms were placed on bacteria seeded NGM plates in triplicate (thirty worms per genotype) and experiments were blinded. The worms were grown at indicated temperature. The worms were switched to new plates every day. The numbers of eggs laid, hatched worms and males were counted. These experiments were performed 2–4 independent times. Paired t-tests were performed to compare mutant brood sizes and two-way ANOVA was performed to examine the interaction between two genotypes.

### Immunofluorescence microscopy

Whole mounted dissected gonads were prepared as described in [60]. Briefly, around 30 worms were picked to 50 μl of azide mix (100 μl 10 X egg buffer, 0.1% Tween-20, 30 μl 0.5 M NaAzide and add H20 to 1 mL) on syliconized slip. Germ lines were extruded by cutting head and tail with #11 surgical blade. Dissected gonads were transferred to a superfrost plus slide on frozen block and transferred directly into cold methanol. The gonads were then post fixed with 4% formaldehyde fix for 30 minutes at room temperature. They were washed with PBST and blocked for 1 hour in 1% BSA. Primary antibody was diluted in PBST and incubated overnight at 4 °C in a humid chamber. Slides were subsequently washed 3 times for 5 minutes each time with PBST before incubation with secondary antibodies for 2 hours at room temperature. The germ lines were washed 3 times and then staining with 1 μg/mL DAPI 10 minutes before being mounted on coverslips with mounting solution. The edges of the slides were then sealed with nail polish. Immunofluorescence images were collected at 0.2 μm intervals with an IX-70 microscope (Olympus) and a CoolSNAP HQ CCD camera (Roper Scientific) controlled by the DeltaVision system (Applied Precision). Images were subjected to deconvolution by using the SoftWoRx 3.3.6 software (Applied Precision). Because the *nmad-1* germlines are smaller than the WT germlines, for RAD-51 quantification the *nmad-1* germline was broken down into 5 equally sized segments as opposed to the 7 segments of a WT germline. The first segment of the *nmad-1* germline was the mitotic region, the second the transition zone, and the third thru fifth were quantified as the meiotic region. In the WT worm germline, the first segment and a half were the mitotic region, one and a half to the third segment constituted the transition zone, and segments four thru seven were quantified as the meiotic region.

### RNAseq

mRNA was extracted from the germlines of 100 worms in each group using Dynabeads mRNA direct micro purification kit (Invitrogen). RNA concentration was measured using Qubit 3.0 and 50 ng of mRNA with an RNA Integrity Number (RIN) > 8, as determined by Agilent BioAnalyzer–RNA Nano. Two biological replicates were assigned for each group, in total 8 groups were prepared for RNA-seq libraries using NEXTflex Illumina qRNA-Seq Library Prep Kit (Bioo Scientific). Briefly, mRNA was fragmented using a cationic buffer. Fragmented RNA underwent first and second strand synthesis, followed by end-repair, 3’- end adenylation and ligation to barcode and pair-end adaptors. Ligated DNA fragments were PCR amplified for 15 cycles. The libraries were cleaned up by Agencourt AmPure XP Magnetic Beads (Beckman Coulter), followed by quantification with a fluorometer Qubit 3.0 and size-check using Agilent 2200 TapeStation D1000. The optimal cluster density for multiplexed libraries was determined by KAPA library quantification kit. The concentration of pooled library (5 nM) was subject to sequencing on NextSeq 500 platform. Pair-end reads of 75 bp were sequenced and trimmed to 50 bp (15 bp at 5’ end and 10 bp at 3’ end) using CutAdapt. All the downstream analyses were based on quality trimmed reads.

The sequencing reads were aligned to the *C*.*elegans* genome (WS220) by bowtie2 and the transcripts were quantified by EBSeq in RSEM pipeline. Transcript and gene expression matrices were built with featurecounts. A script in trinity toolkit “PtR” was used to explore the correlation between biological replicates, and generate principal component analysis (PCA) and heatmap among the sample replicates. For calling the significant differentially expressed genes (DEGs), the false discovery rate (FDR) after multiple testing correction was set as 0.05 and analyzed in edgeR. Gene ontology was performed in DAVID and R package–ClusterProfiler (version 3.9.1). The raw data is uploaded onto GEO (accession number GSE112488).

### Acridine orange staining

Age-matched (24 hours post-L4) worms were analyzed by acridine orange staining as described in [61]. Briefly, worms were incubated with 500 μl M9 buffer containing 0.02 mg/mL acridine orange in the dark at room temperature for 1 hour with gentle shaking. The stained worms were transferred to regular NGM plates and kept in the dark so that the worms recovered and de-stained for 1 hour. Worms were then mounted on slides and analyzed by fluorescence microscopy. Between 15 and 20 gonads were scored for each genotype and the experiment was performed in triplicate utilizing a Leica DM5000B fluorescence microscope.

### Immunoprecipitation/mass spectrometry

Mixed stage worms were washed off plates with M9 buffer. Worms were washed 5 times in M9 buffer and snap frozen in liquid N2 six times. Samples were centrifuged and pellet was resuspended in low-salt buffer (20 mM Tris pH 7.5, 50 mM KCl, 0.4 mM EDTA, 5 mM MgCl2, 10% glycerol, 0.1% Triton-X100, 1 mM B-mercaptoethanol, 1 mM PMSF). Worms were homogenized by douncing with a glass homogenizer. After homogenization, the cell extract was centrifuged at 50,000 xg for 30 minutes at 4 °C and the supernatant was discarded. The pellet was resuspended in high-salt buffer (20 mM HEPES pH 7.0, 200 mM NaCL, 5 mM MgCl2, 10% glycerol, 0.1% Triton-X100, 1mM B-mercaptoethanol, 1mM PMSF) and sonicated for 12 cycles of 20 seconds of sonication followed by 40 seconds of rest at 4 °C. Samples were centrifuged and the supernatant was collected and incubated for 3 hours at 4 °C with Protein A Dynabeads (ThermoScientific) conjugated to antibodies. The beads were washed with high-salt buffer 5 times followed by elution with 0.1 M glycine (pH 2.5). 10% of the sample was run out on SDS-page gel and silver stained and the remainder was submitted to the Taplin Mass Spectrometry Facility for analysis.

### *In vitro* binding assays

*In vitro* binding assays were performed to validate the interactions between NMAD-1and candidate interacting proteins. The coding sequence of NMAD-1 was cloned in HIS tagged vector pET28 and the coding sequences of candidate proteins were cloned separately as in-frame fusion to the GST tagged vector pGEX-4T1. The recombinant proteins were expressed in *E*. *coli* BL21. Overnight induction of protein expression was carried out with 1 mM IPTG at 18 °C. Bacteria were harvested at 4000 rpm, 4°C and 10 mL protein purification lysis buffer (50 mM pH7.5 Tris-HCl, 0.25 M NaCl, 0.1% Triton-X, 1 mM PMSF, 1 mM DTT, and protease inhibitors. For HIS purification, 20 mM imidazole was added. After freezing the pellet at -80°C for 1 hour, the lysate was sonicated with a Bioruptor for 5 minutes on high level with 30s on and 30s off. Proteins were purified with glutathione sepharose 4B (for GST) or Ni-NTA (for HIS) beads. Proteins and beads were washed 3 times with protein purification lysis buffer before incubating the beads with elution buffer (12 mg/ml Glutathione in protein purification lysis buffer for GST protein elution, pH 8 and 400mM Imidazole in protein purification lysis buffer for HIS protein elution) for 30 minutes. Eluates were dialyzed overnight at 4 °C with dialysis buffer (50 mM pH7.5 Tris-HCl, 100 mM KCl, 5 mM MgCl2, 0.1% NP-40, 10% Glycerol, 1 mM PMSF and 1 mM DTT). Bradford assays and SDS-page gel electrophoresis followed by coomassie staining was performed to determine integrity and quantity of purified proteins. For each binding assay, combined 30 μl blocked glutathione sepharose 4B beads, 3 μg GST-tagged protein and 1 μg HIS-tagged NMAD-1 in 500 μl TAP wash buffer (50 mM pH 8.0 Tris-HCl, 100 mM KCl, 10% glycerol, 5 mM MgCl2, 0.2 mM EDTA, 0.1% NP-40). Samples were rotated at 4 °C for 1 hour to bind. Then samples were washed 3 times with 1 mL TAP wash buffer and 1 time with 1 mL PBS. 20 μl of 2X SDS loading buffer was added and samples were boiled at 95 °C for 10 minutes. Samples were then analyzed by SDS-PAGE and western blot.

### *In vivo* binding assay

Worms were washed with M9 buffer 3 times and then pelleted. The pellet was frozen and thawed in liquid nitrogen 6 times and then homogenization on ice for 10 minutes. Then 1ml IP-200 buffer (20 mM pH7.5 Tris-HCl, 50 mM KCl, 0.4 mM EDTA, 5 mM MgCl2, 10% Glycerol, 0.1% Triton-X100, 1 mM β-Me, 1 mM PMSF and proteinase inhibitor cocktail) was added to the worm pellet and sonicated 2 times with a Bioruptor, for 5 minutes each time, at 30s on and 30s off. Samples were then centrifuged at 14,000 rpm at 4 °C for 10 minutes. The supernatant was transferred to a new tube and antibody was added before rotating at 4 °C for 2 hours. Beads were blocked with BSA. Blocked beads were added to the IP sample and rotated for 2 hours at 4 °C. Samples were washed with 1mL IP-200 buffer 5 times and 1 time with PBS. 20 μl of 2X SDS loading buffer was added and then samples were boiled at 95 °C for 10 minutes. Samples were analyzed by SDS-PAGE and western blot.

### EdU feeding

*E*. *coli* strain MG1693 was grown overnight at 37°C in M9 minimal media (3 g/L KH2PO4, 6 g/L Na2HPO4, 0.5 g/L NaCl, 1 g/L NH4Cl, 2 mM MgSO4, 0.1 mM CaCl2, 0.4% glucose, and 1 μg/ml thiamin) supplemented with 5 μg/ml thymine. This culture was diluted 1:50 in M9 minimal media supplemented with 0.5 μM thymidine and 20 μM EdU and grown for 36 hours at 37°C. Harvested cells were re-suspended in 1/100 of their original volume in M9, and then spread onto 15 cm Edu labeling plates (standard nematode growth media except that peptone was omitted and agar was 12 g/L agar, 6 g/L agarose), using 1ml of *E*. *coli* solution per plate. L4 staged worms were transferred to plates for defined period of time. After labeling, gonads were dissected and fixed, then incubated with Click-it Edu Imaging Kit (Invitrogen) and DAPI at 0.5 μg/mL. The number of Edu positive and the total number of nuclei were scored.

### Hydroxyurea (HU) treatment

Stage-matched P0 generation L4 worms of WT, *nmad-1* and D186A catalytic mutants were plated at 25 °C, and the L4 worms of subsequent F1 generation were plated on control or 15 mM HU plates for 24 hours treatment. The gonad arms from young adults were dissected and stained by DAPI. Images were collected at 0.2 μm intervals with an IX-70 microscope (Olympus) and a CoolSNAP HQ CCD camera (Roper Scientific). The diameters for the nuclei were measured by Fiji (Version 2.2.0-rc-68/1.52e).

### ChIPseq

L4 stage worms were collected at 20°C and 25°C. The worms were crosslinked with 1x linking buffer (11 mM HEPES-NaOH, 110 mM NaCl, 1.1 mM EDTA and 1.1 mM EGTA) with 1% formaldehyde for 10 minutes at RT. The reaction was quenched by adding glycine to 0.125 M final concentration for 5 minutes at RT. Samples were washed with cold PBS twice. The samples were next dissociated with lysis buffer and sonicated by biorupter for 25 cycles in the cold room with 30 seconds on and 30 seconds off per cycle. Protein G beads were coupled to the flag antibody (F3165 Sigma) for 2 hours. Uncoupled Protein G beads were incubated with the sonicated samples to pull down non-specific binding chromatin. The precleared chromatin samples were incubated with antibody-coupled bead slurries and rotated at 4°C overnight. The bound DNA was eluted from the beads by 200 μl TE with 1% SDS after washing with low salt (0.2% SDS, 2% Triton X-100, 4 mM EDTA, 40 mM Tris-HCl), high salt (0.2% SDS, 2% Triton X-100, 4 mM EDTA, 40 mM Tris-HCl, and 1 M NaCl) and LiCl buffers (0.5 M LiCl, 2% NP-40, 1% Na deoxycholate, 2 mM EDTA, and 20 mM and Tris-HCl). The eluted DNA was next purified by phenol-chloroform. For the library preparation, NEBNext DNA library prep master mix set for Illumina (E6040L) was used per the company’s instructions. Briefly, the fragmented DNA was subjected to end repair, A-tailing, adaptor ligation, PCR enrichment and AMPure XP bead clean up after each of these steps. The libraries were quantified by Qubit 3 fluorometer and the fragments were analyzed by the Agilent D1000 ScreenTape system. The quantified libraries were sequenced by NextSeq 500 and approximately 400 million reads were generated. The raw fastq files were quality checked through Fastqc(0.11.5) and the adaptors were trimmed through Trimmomatic(0.36). The trimmed reads were mapped to Bowtie2(2.2.9) with ce10 build indexes. The unique mapping reads were selected with MAPQ value greater than 10 by samtools (1.3.1). The sorted bam files were deduplicated and peaks were called with Picard (2.8.0) and Macs2(2.7.12) with *p* = 0.05, respectively. The common peaks from biological replicates were identified through Irreproducibility Discovery Rate (IDR 2.0.2). The blacklist regions and non-specific antibody binding regions were removed by bedtools (2.26.0). The raw data is uploaded onto GEO (accession number GSE112488).

## Supporting information

S1 Fig *nmad-1* increased incidence of males depends on the catalytic domain and COSA-1 staining is normal despite defects in Prophase I. A) D186A transgenic worms have an increased incidence of males when cultured at 25°. Graph represents the mean ± SEM of seven independent experiments of ten worms per genotype performed in triplicate. B) COSA-1 marks 6 distinct foci per nucleus in both WT and *nmad-1* mutant worms at the late pachytene to diplotene stage of oocyte maturation. A GFP::COSA-1 transgenic strain was crossed into *nmad-1* mutant strains to observe COSA-1 foci. DAPI is shown in blue while GFP::COSA-1 is shown in green. Representative images are shown in the left panel and quantification of 31–35 nuclei is shown in the right panel. C) COSA-1 marks six distinct foci in both WT and *nmad-1* mutant worms at the diakinesis stage. Shown are representative images of -2 oocytes (position is relative to the spermatheca). DAPI is shown in blue while GFP::COSA-1 is shown in green.
(TIF)

S2 Fig Misregulated genes and processes in *nmad-1* mutant worms. A) MA plots show significantly misregulated genes (represented as red dots) at 20° and 25° of extracted germlines from WT and *nmad-1* mutant worms. B) Principal component analysis of RNAseq datasets demonstrates that WT and *nmad-1* gene expression are more similar at 20° than at 25° and that replicate datasets cluster together. The top gene ontology (GO) categories of C) up regulated or D) down regulated genes in *nmad-1* mutant worms at 25° relative to the genome. The top gene ontology (GO categories of E) up regulated or F) down regulated genes in *nmad-1* mutant worms at 25°C relative to germline expressed genes [38] are enriched for genes regulating reproduction, apoptosis, DNA replication, and DNA repair.
(TIF)

S3 Fig *nmad-1* mutant worms have a developmental delay. Percentage of worms in each developmental stage (red: eggs, orange: L1, yellow: L2, green: L3, blue: L4, purple: adult) when cultured at A) 20° or at B) 25°. Graphs represent a representative experiment of four independent experiments performed by two researchers performed in sextuplicate ± SD.
(TIF)

S4 Fig Increased DNA damage is SPO-11-dependent and resolved by the diakinesis stage. A) RAD-51 staining is unobservable in WT or *nmad-1* mutant worms at the diakinesis stage (left panel) or in early (upper right panel) or late eggs (lower right panel). B) Quantification of RAD-51 foci in 4–8 germlines from WT, *nmad-1*, *spo-11*, *nmad-1;spo-11* mutant worms demonstrate increased SPO-11-dependent foci arising earlier, being more plentiful, and persisting longer in *nmad-1* mutant worms. C) *nmad-1* mutant worms have abnormal chromosome number and compaction at the diakinesis stage at 25°C as quantified by DAPI staining for DNA while *spo-11* mutant worms always have 12 univalents at the diakinesis stage regardless of *nmad-1* genotype. Representative images are shown above on the right panel and a compilation of 23–47 nuclei are quantified in the lower panel. ns: not significant, **** p<0.0001. D) There is increased apoptosis in the *nmad-1* mutant germline as assessed by CED-1::GFP fluorescence. Yellow arrows point to apoptotic nuclei. Staining in left panels and differential interference contrast images (DIC) shown in right panels. Images were taken of the gonadal loop region as developing oocytes transition from the pachytene to the diplotene stage. E) Increased apoptosis in *nmad-1* mutant worms is dependent on the p53 homolog *cep-1* and the caspase 1 homolog *ced-3* as assessed by quantification of apoptosis occurrence in WT, *nmad-1*, *cep-1*, *cep-1*:*nmad-1*, *ced-3*, and *nmad-1;ced-3* mutant worms by CED-1::GFP fluorescence. These graphs represent the mean ± SEM of three independent experiments: each experiment consists of apoptosis measurements of 15–20 worm germlines per genotype examined at 25°. Individual genotypes were compared by paired t tests while the interaction of genotypes was analyzed by two-way ANOVA. ns: not significant, * p<0.05, ** p<0.01.
(TIF)

S5 Fig Confirmation of direct interaction of NMAD-1 with some proteins identified by mass spectrometry by expression in bacterial BL21 cells. Co-expression of a His-tagged NMAD-1 and putative NMAD-1 binding proteins identified in Fig 4A in BL21s followed by His pull down experiments reveals that NMAD-1 interacts directly with CEH-93, MCM-4, and F37C4.5. The starred bands are consistent with the appropriate molecular weight of CEH-93, MCM-4, and F37C4.5 and no additional validation was performed.
(TIF)

S6 Fig The number of nuclei entering M phase was similar among wild type and *nmad-1* mutant worms. A) Representative image shows 5–8 nuclei entering M phase in different groups. DAPI is shown in blue and Histone 3 Serine 10 phosphorylation is shown in red. B) Quantification of the ratio of M phase nuclei number to the total nuclei number at progenitor zones. Each bar represents the mean ± SD of 10 germlines.
(TIF)

S1 Table RNAseq and gene ontology analysis of extracted germlines from wild type and *nmad-1* mutant worms grown at 20° and 25°. (XLSX)

S2 Table Mass spectrometry analysis of proteins bound to GFP::3xFlag::NMAD-1. (XLSX)

S3 Table Analysis of chromatin immunoprecipitation followed by sequencing (ChIPseq) with a Flag antibody of *top-2*::*3xflag*, *gfp*::*3xflag*::*nmad-1*, and *top-2*::*3xflag;nmad-1* transgenic strains. (XLSX)
