# Supplementary figures and images for: The demethylase NMAD-1 regulates DNA replication and repair in the Caenorhabditis elegans germline
Source: PLoS Genet. 2019 Jul 8;15(7):e1008252. doi: 10.1371/journal.pgen.1008252 (PMC6638966; doi:10.1371/journal.pgen.1008252)

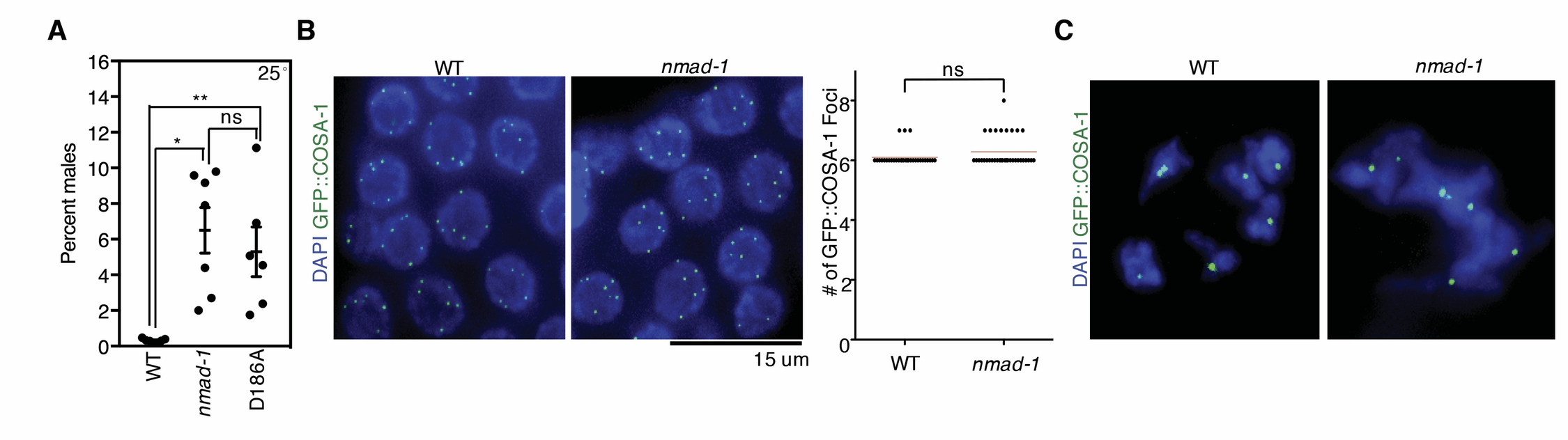

Supplement: S1 Fig — A) D186A transgenic worms have an increased incidence of males when cultured at 25°. Graph represents the mean ± SEM of seven independent experiments of ten worms per genotype performed in triplicate. B) COSA-1 marks 6 distinct foci per nucleus in both WT and nmad-1 mutant worms at the late pachytene to diplotene stage of oocyte maturation. A GFP::COSA-1 transgenic strain was crossed into nmad-1 mutant strains to observe COSA-1 foci. DAPI is shown in blue while GFP::COSA-1 is shown in green. Representative images are shown in the left panel and quantification of 31–35 nuclei is shown in the right panel. C) COSA-1 marks six distinct foci in both WT and nmad-1 mutant worms at the diakinesis stage. Shown are representative images of -2 oocytes (position is relative to the spermatheca). DAPI is shown in blue while GFP::COSA-1 is shown in green. (TIF) [file pgen.1008252.s001.tif]

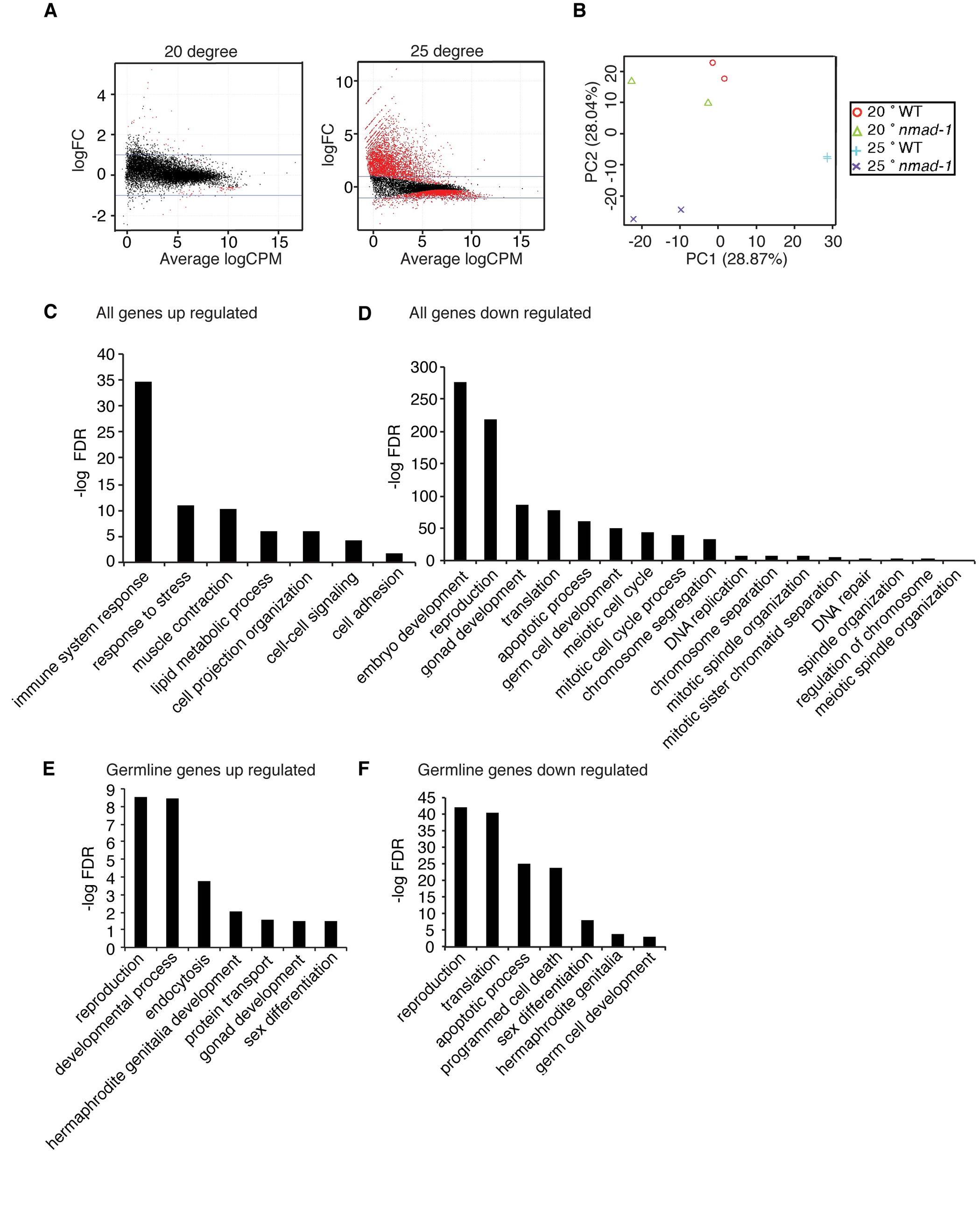

Supplement: S2 Fig — A) MA plots show significantly misregulated genes (represented as red dots) at 20° and 25° of extracted germlines from WT and nmad-1 mutant worms. B) Principal component analysis of RNAseq datasets demonstrates that WT and nmad-1 gene expression are more similar at 20° than at 25° and that replicate datasets cluster together. The top gene ontology (GO) categories of C) up regulated or D) down regulated genes in nmad-1 mutant worms at 25° relative to the genome. The top gene ontology (GO categories of E) up regulated or F) down regulated genes in nmad-1 mutant worms at 25°C relative to germline expressed genes [38] are enriched for genes regulating reproduction, apoptosis, DNA replication, and DNA repair. (TIF) [file pgen.1008252.s002.tif]

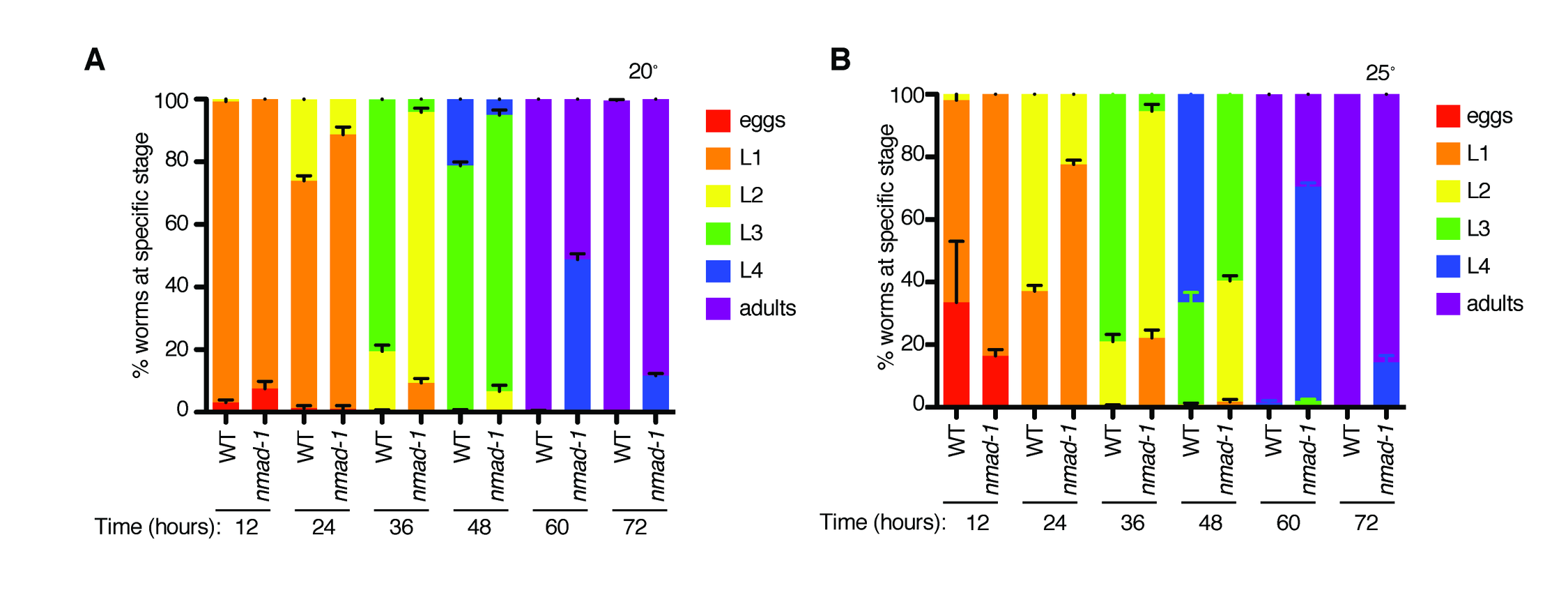

Supplement: S3 Fig — Percentage of worms in each developmental stage (red: eggs, orange: L1, yellow: L2, green: L3, blue: L4, purple: adult) when cultured at A) 20° or at B) 25°. Graphs represent a representative experiment of four independent experiments performed by two researchers performed in sextuplicate ± SD. (TIF) [file pgen.1008252.s003.tif]

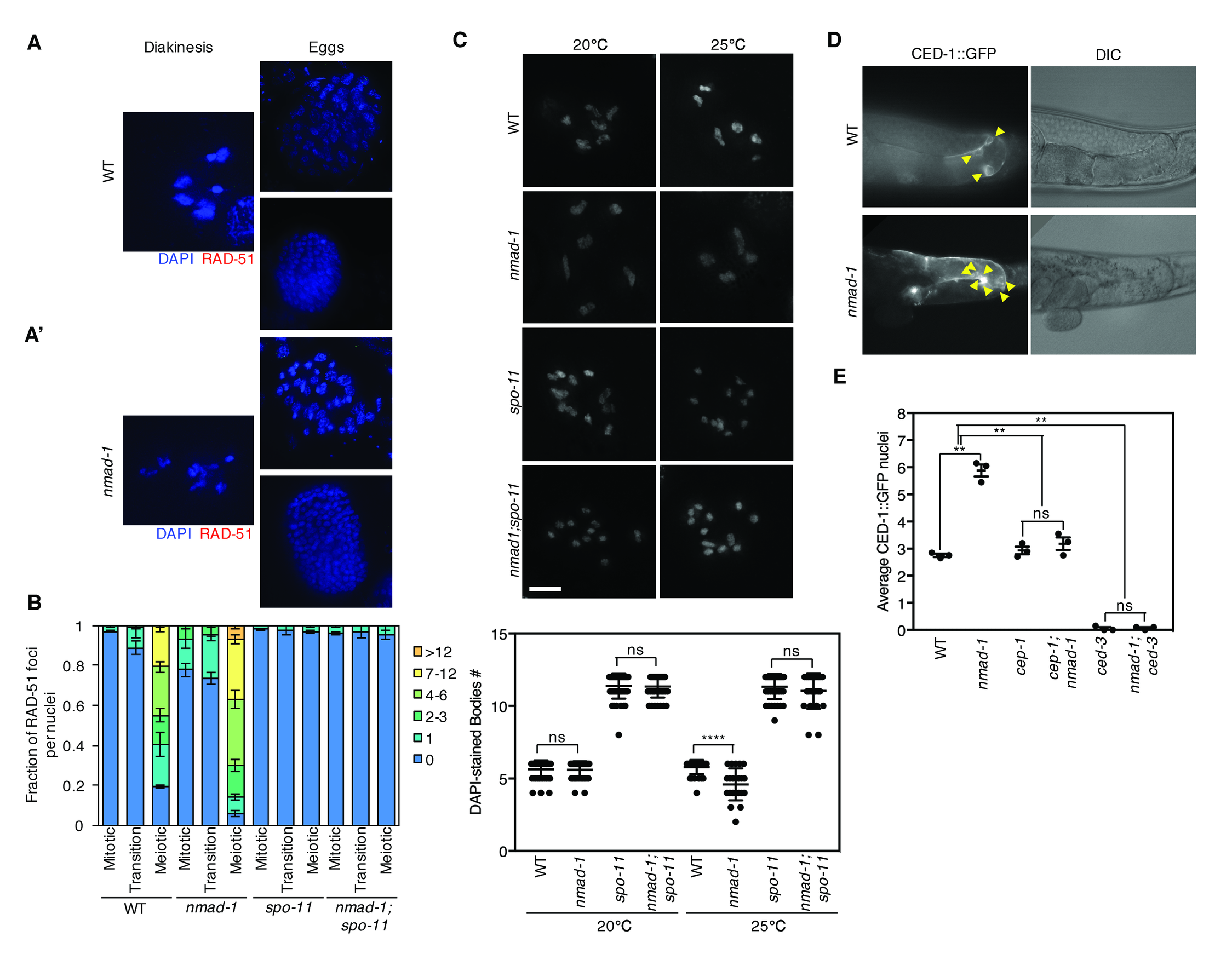

Supplement: S4 Fig — A) RAD-51 staining is unobservable in WT or nmad-1 mutant worms at the diakinesis stage (left panel) or in early (upper right panel) or late eggs (lower right panel). B) Quantification of RAD-51 foci in 4–8 germlines from WT, nmad-1, spo-11, nmad-1;spo-11 mutant worms demonstrate increased SPO-11-dependent foci arising earlier, being more plentiful, and persisting longer in nmad-1 mutant worms. C) nmad-1 mutant worms have abnormal chromosome number and compaction at the diakinesis stage at 25°C as quantified by DAPI staining for DNA while spo-11 mutant worms always have 12 univalents at the diakinesis stage regardless of nmad-1 genotype. Representative images are shown above on the right panel and a compilation of 23–47 nuclei are quantified in the lower panel. ns: not significant, **** p<0.0001. D) There is increased apoptosis in the nmad-1 mutant germline as assessed by CED-1::GFP fluorescence. Yellow arrows point to apoptotic nuclei. Staining in left panels and differential interference contrast images (DIC) shown in right panels. Images were taken of the gonadal loop region as developing oocytes transition from the pachytene to the diplotene stage. E) Increased apoptosis in nmad-1 mutant worms is dependent on the p53 homolog cep-1 and the caspase 1 homolog ced-3 as assessed by quantification of apoptosis occurrence in WT, nmad-1, cep-1, cep-1:nmad-1, ced-3, and nmad-1;ced-3 mutant worms by CED-1::GFP fluorescence. These graphs represent the mean ± SEM of three independent experiments: each experiment consists of apoptosis measurements of 15–20 worm germlines per genotype examined at 25°. Individual genotypes were compared by paired t tests while the interaction of genotypes was analyzed by two-way ANOVA. ns: not significant, * p<0.05, ** p<0.01. (TIF) [file pgen.1008252.s004.tif]

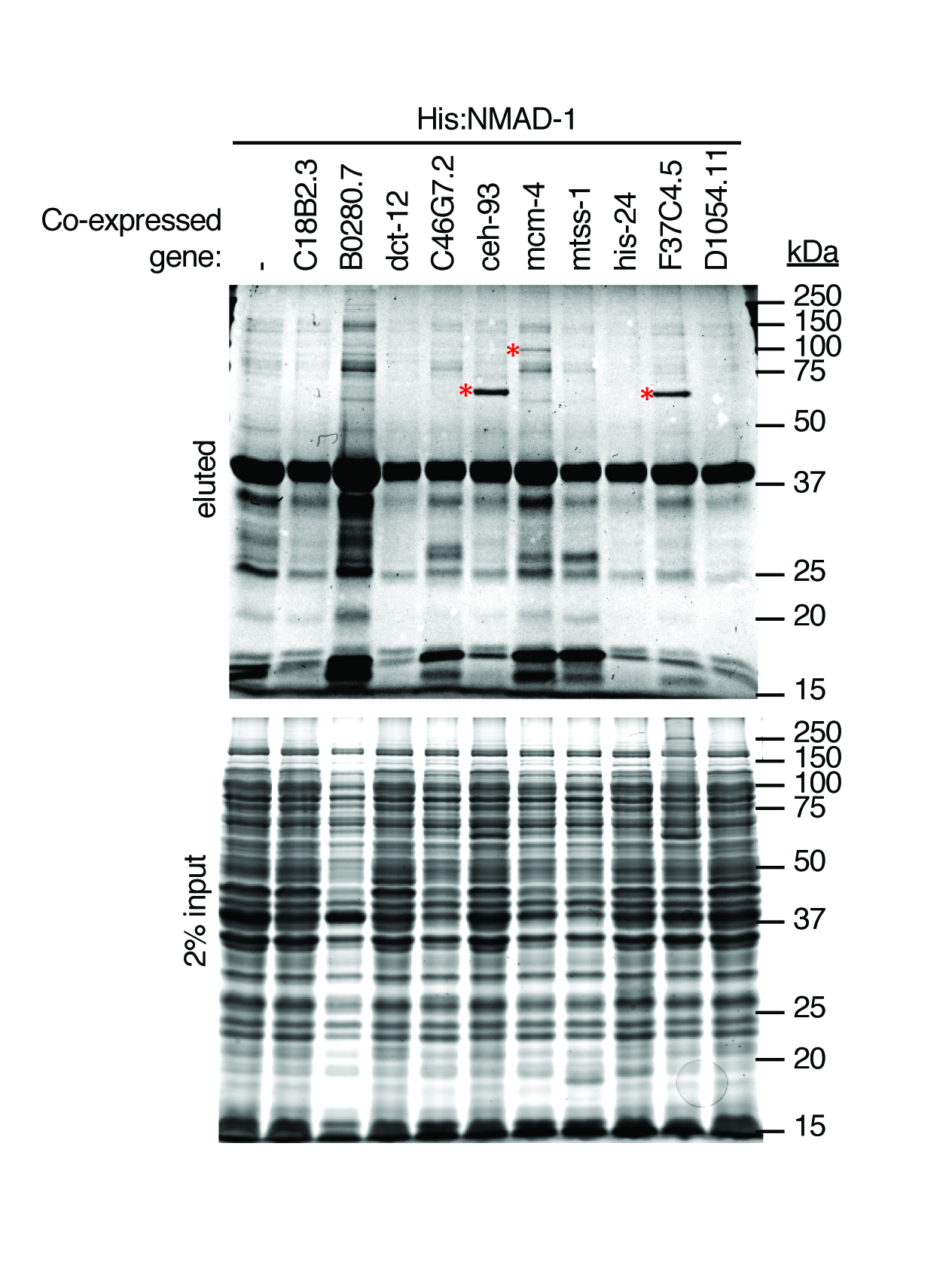

Supplement: S5 Fig — Co-expression of a His-tagged NMAD-1 and putative NMAD-1 binding proteins identified in Fig 4A in BL21s followed by His pull down experiments reveals that NMAD-1 interacts directly with CEH-93, MCM-4, and F37C4.5. The starred bands are consistent with the appropriate molecular weight of CEH-93, MCM-4, and F37C4.5 and no additional validation was performed. (TIF) [file pgen.1008252.s005.tif]

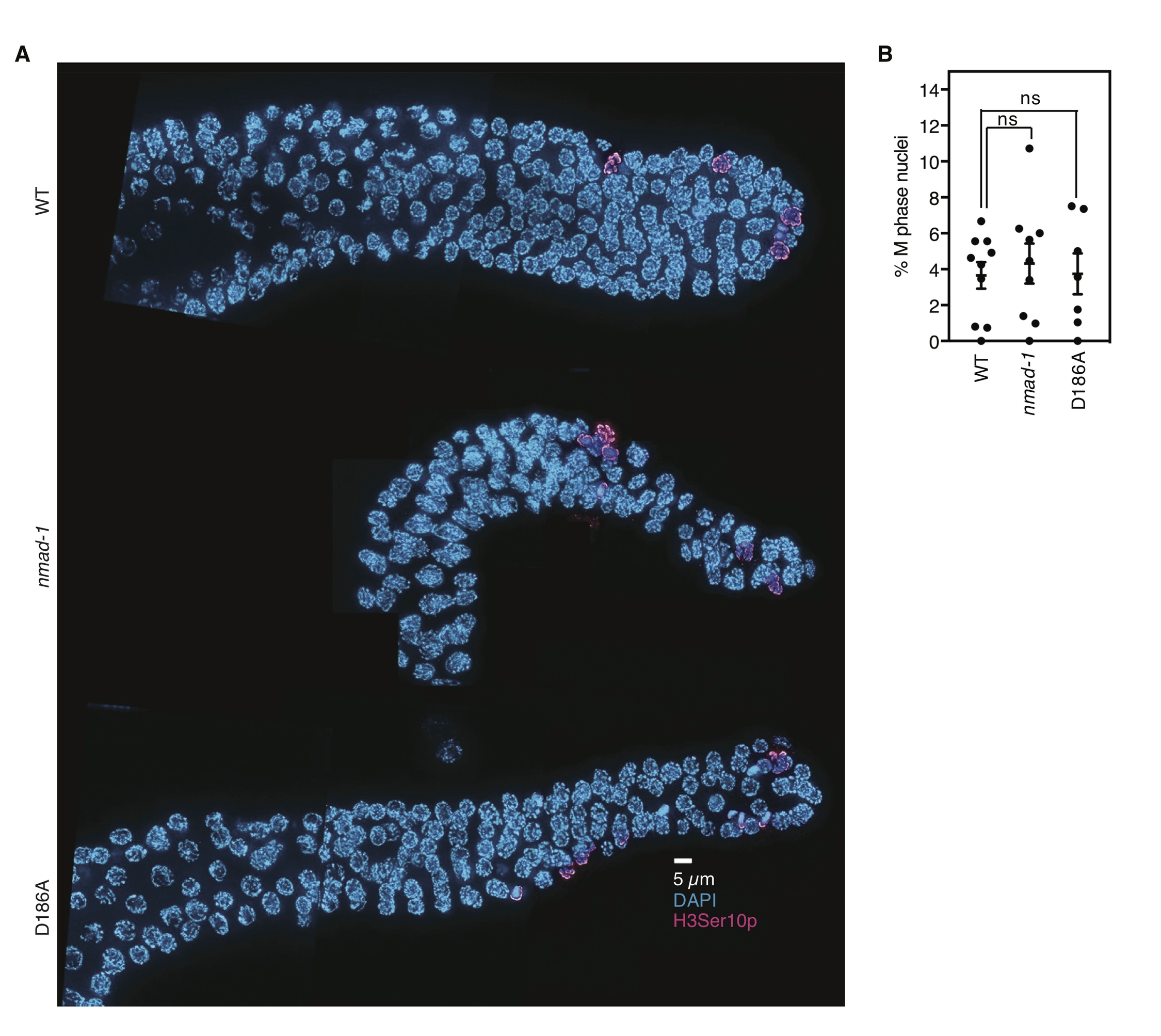

Supplement: S6 Fig — A) Representative image shows 5–8 nuclei entering M phase in different groups. DAPI is shown in blue and Histone 3 Serine 10 phosphorylation is shown in red. B) Quantification of the ratio of M phase nuclei number to the total nuclei number at progenitor zones. Each bar represents the mean ± SD of 10 germlines. (TIF) [file pgen.1008252.s006.tif]
